# The influence of habitual consumption of alcohol on the incidence of obstructive sleep apnea: a national population-based cohort study

**DOI:** 10.1007/s11325-026-03684-1

**Published:** 2026-04-18

**Authors:** Jina Park, Eunhye Bae, Yong Jin Sim, Jaeyoung Cho

**Affiliations:** 1 Division of Pulmonary and Critical Care Medicine, Department of Internal Medicine, Seoul National University Hospital, 101 Daehak-ro, Jongno-gu, Seoul, 03080 Republic of Korea; 2 Division of Pulmonary and Critical Care Medicine, Department of Internal Medicine, Chung-ang University Gwangmyeong Hospital, Gyeonggi-do, Republic of Korea; 3 Department of Biostatistics and Computing, Yonsei University, Seoul, Republic of Korea; 4 Department of Internal Medicine, Seoul National University College of Medicine, Seoul, Republic of Korea

**Keywords:** Obstructive sleep apnea, Alcohol consumption, Incidence, Risk factors

## Abstract

**Purpose:**

The objective of this nationwide population-based cohort study was to determine the association between habitual alcohol consumption and the incidence of newly diagnosed obstructive sleep apnea (OSA).

**Methods:**

This study used data from the Korean National Health Insurance Service database and included individuals aged ≥ 40 years who underwent a health check-up in 2011 or 2012. Individuals with a prior history of sleep apnea were excluded. Habitual alcohol consumption was defined as a weekly alcohol intake greater than 0 g. The study outcome was the newly identified cases of OSA, which we evaluated using the Cox proportional hazards regression model.

**Results:**

A total of 3,988,993 individuals (mean age, 54.5 years; male, 50.1%; mean body mass index [BMI], 24.0 kg/m²) were included, of whom 1,688,094 (42.3%) had habitual alcohol consumption. During a mean follow-up period of 9.7 years, 33,563 new cases of OSA were documented. Individuals with habitual alcohol consumption had an incidence rate of 108.9 per 100,000 person-years, whereas those without habitual alcohol consumption had a rate of 69.6 per 100,000 person-years. Following adjustment for covariates including age, sex, BMI, income, smoking status, physical activity, and the Charlson comorbidity index, the hazard ratio for incident OSA was 1.026 (95% confidence interval, 1.001–1.051). The association between habitual alcohol consumption and incident OSA was more prominent among males and those with underlying chronic kidney disease or a history of cancer.

**Conclusion:**

This large-scale Korean population-based cohort study revealed that habitual alcohol consumption is associated with an increased incidence of OSA.

**Supplementary Information:**

The online version contains supplementary material available at 10.1007/s11325-026-03684-1.

## Introduction

Obstructive sleep apnea (OSA) is a common disorder characterized by episodic collapse of the upper airway during sleep, resulting in chronic intermittent hypoxia, sleep fragmentation, and excessive daytime sleepiness [1]. Globally, it is estimated that approximately 1 billion adults aged 30–69 years are affected by OSA, and 425 million are affected by moderate to severe disease [2]. OSA is associated with a wide range of adverse health outcomes, including cardiovascular diseases, metabolic dysfunction, neurocognitive impairment, and increased mortality [1, 3]. Moreover, OSA poses a substantial societal burden, particularly due to excessive daytime sleepiness, which increases the risk of motor vehicle accidents and workplace injuries [4].

Alcohol consumption has been recognized as a modifiable behavioral factor that may influence the incidence of OSA [5, 6]. Alcohol impairs upper airway muscle tone, increases upper airway collapsibility, and suppresses arousal responses, which can exacerbate the frequency and severity of apneic events during sleep [7, 8]. Several observational studies have reported a positive association between alcohol consumption and increased risk or severity of OSA [5, 8, 9]. A meta-analysis concluded that alcohol consumption increased the risk of OSA by 25% [6]. However, these findings were mostly from cross-sectional or case-control studies [10], which are limited in establishing temporal relationships and susceptible to recall and selection biases. In addition, recent Mendelian randomization studies failed to show a significant association between alcohol consumption and OSA [11, 12].

Our scoping of the literature identified a need for a large-scale longitudinal population-based study to evaluate the relationships between habitual alcohol consumption and the incidence of OSA. Accordingly, we conducted a nationwide population-based cohort study using data from the Korean National Health Insurance Database (NHID). We hypothesized that habitual alcohol consumption is independently associated with an increased incidence of newly diagnosed OSA. The significance of this research lies in clarifying the temporal relationship between alcohol use and OSA development within a large population and identifying high-risk subgroups for targeted clinical intervention.

## Methods

### Study design and population

The study utilized a nationwide population-based cohort design, following habitual and non-habitual alcohol consumers between 2011/2012 and 2021 to evaluate the association between habitual alcohol consumption and the incidence of OSA. This study used data from the Korean NHID. The NHID, maintained by the National Health Insurance Service (NHIS), is a representative big data repository that covers the entire South Korean population (approximately 51 million individuals) [13, 14]. As South Korea operates a single-payer healthcare system, the NHIS provides universal coverage to 97% of the population through the National Health Insurance program and to the remaining 3% of low-income individuals through the medical aid program [14]. The NHID encompasses a wide range of health-related information, including demographic data, medical diagnoses, procedures, prescriptions, healthcare utilization, and lifestyle factors such as smoking status, alcohol consumption, and physical activity [15]. In addition, the NHIS provides biennial health check-ups to adults aged 20 years and older, with a participation rate of 75% [13, 14].

Our study included individuals aged ≥ 40 years who underwent a health checkup in 2011 or 2012 and had no prior history of sleep apnea (recorded as G47.3 in the 10th revision of the International Classification of Diseases [ICD-10]) from 2002 until the index date (the date when participants underwent the health checkup). We excluded the following individuals: (1) those with incident sleep apnea within 12 months following the index date, (2) those with incident central sleep apnea or unspecified sleep apnea occurring more than 12 months after the index date, and (3) those with missing or incomplete data. Individuals diagnosed with OSA within the first 12 months were excluded to minimize the possibility of reverse causality and to ensure a clearer temporal relationship between alcohol consumption and subsequent disease occurrence.

This study was exempted from ethical review by the Institutional Review Board of Seoul National University Hospital (E-2402-016-1506), as all data were anonymized, and it was conducted in accordance with the tenets of the Declaration of Helsinki.

### Variables and outcome

Habitual alcohol consumption was defined as weekly alcohol intake greater than 0 g based on the self-reported questionnaire [16]. The questionnaire consisted of two questions assessing current status: “On average, how many days per week do you drink alcohol?” and “How much do you usually drink per day when you drink (in the number of glasses)?” [17] One can of beer (355 cc) is equivalent to 1.6 glasses of beer. A standard drink is considered to contain approximately 7.5 g of alcohol [17].

We obtained the following data on the index date from the NHID: age, sex, anthropometric measurements (height, weight, body mass index [BMI], and waist circumference), income quartile, smoking status, physical activity, comorbidities, blood pressure, and laboratory findings. According to the World Health Organization and Asian-Pacific guidelines, obesity was defined as a BMI of 25 or higher [18]. Central obesity was defined as a waist circumference ≥ 90 cm in men and ≥ 85 cm in women [19]. Smoking status was categorized as never, former, or current smokers. Never-smokers were defined as individuals who had smoked fewer than 100 cigarettes in their lifetime, while the remaining individuals were classified as either former or current smokers based on their current smoking status. Physical activity was assessed using questionnaires regarding the frequency (days per week) of vigorous-intensity physical activity lasting at least 20 min, moderate-intensity physical activity lasting at least 30 min, and light-intensity physical activity lasting at least 30 min. Weekly metabolic equivalent of task (MET) scores were calculated, with ratings of 3.0, 4.0, and 8.0 METs assigned to light-intensity, moderate-intensity, and vigorous-intensity physical activities, respectively [20]. Then, the physical activity levels were categorized into four groups: inactive (0 MET-min/wk), insufficiently active (1–499 MET-min/wk), active (500–999 MET-min/wk), and highly active ($\:\ge\:$1,000 MET-min/wk) [20]. Additionally, moderate to vigorous physical activity (MVPA) was categorized based on weekly frequency into four groups: inactive, 1–2 times per week, 3–4 times per week, and 5 or more times per week [21]. The following comorbidities were defined by their respective ICD-10 codes: hypertension was identified with codes I10 through I13 and also I15; type 2 diabetes with codes E11, E12, E13, and E14; ischemic heart disease with codes from I20 up to I25; and heart failure using I50. Transient ischemic attack and cerebral infarction were classified using G45 and I63, while atrial fibrillation or flutter was captured under I48. Chronic obstructive pulmonary disease (COPD) was defined by J44, cancer by codes ranging from C00 to C97, and fatty liver by K70.0 and K76.0. Chronic kidney disease (CKD) was defined as an estimated glomerular filtration rate (eGFR) of < 60 mL/min/1.73 m², calculated using the CKD epidemiology collaboration equation [22]. When the eGFR was missing, ICD-10 codes N18–N19, Z49, and Z99.2 were used to define CKD [14]. The Charlson comorbidity index (CCI) was also calculated [23].

The main outcome of the present study was the diagnosis of OSA, identified using ICD-10 code G47.33. Individuals were followed up from the index date until the earliest occurrence of diagnosis of OSA, death, or the study endpoint (December 31, 2021).

### Statistical analyses

Categorical variables were summarized as frequencies and percentages, while continuous variables were reported as means with standard deviations (SD) or medians with interquartile ranges (IQR). Baseline characteristics between individuals with and without habitual alcohol consumption were analyzed using either Student’s *t*-tests (for normally distributed continuous variables) or Mann–Whitney *U* tests (for non-normally distributed continuous variables) and χ2 or Fisher’s exact tests for categorical variables. Statistical analyses focused on comparing outcomes between the two main groups: individuals with habitual alcohol consumption and individuals with non-habitual alcohol consumption (weekly alcohol intake of 0 g). Incidence rates of OSA were calculated as the number of newly diagnosed cases per 100,000 person-years. A Cox proportional hazards model was used to evaluate the association between alcohol consumption and the incidence of OSA, adjusting for age, sex, BMI, quartile of income, smoking status (never, former, or current), physical activity (inactive, insufficiently active, active, or highly active), and CCI. The risks of OSA are expressed as hazard ratios (HRs) with 95% confidence intervals (CIs). We also performed subgroup analyses, stratified by age, sex, income, BMI, central obesity, smoking status, physical activity, CCI, and various comorbidities. All comparisons were two-sided, with a significance threshold set at *P* < 0.05. Statistical analyses were conducted using R (version 4.3.3) and SAS version 9.4 (SAS Institute, Cary, NC, USA).

## Results

### Baseline characteristics

**Fig. 1:**
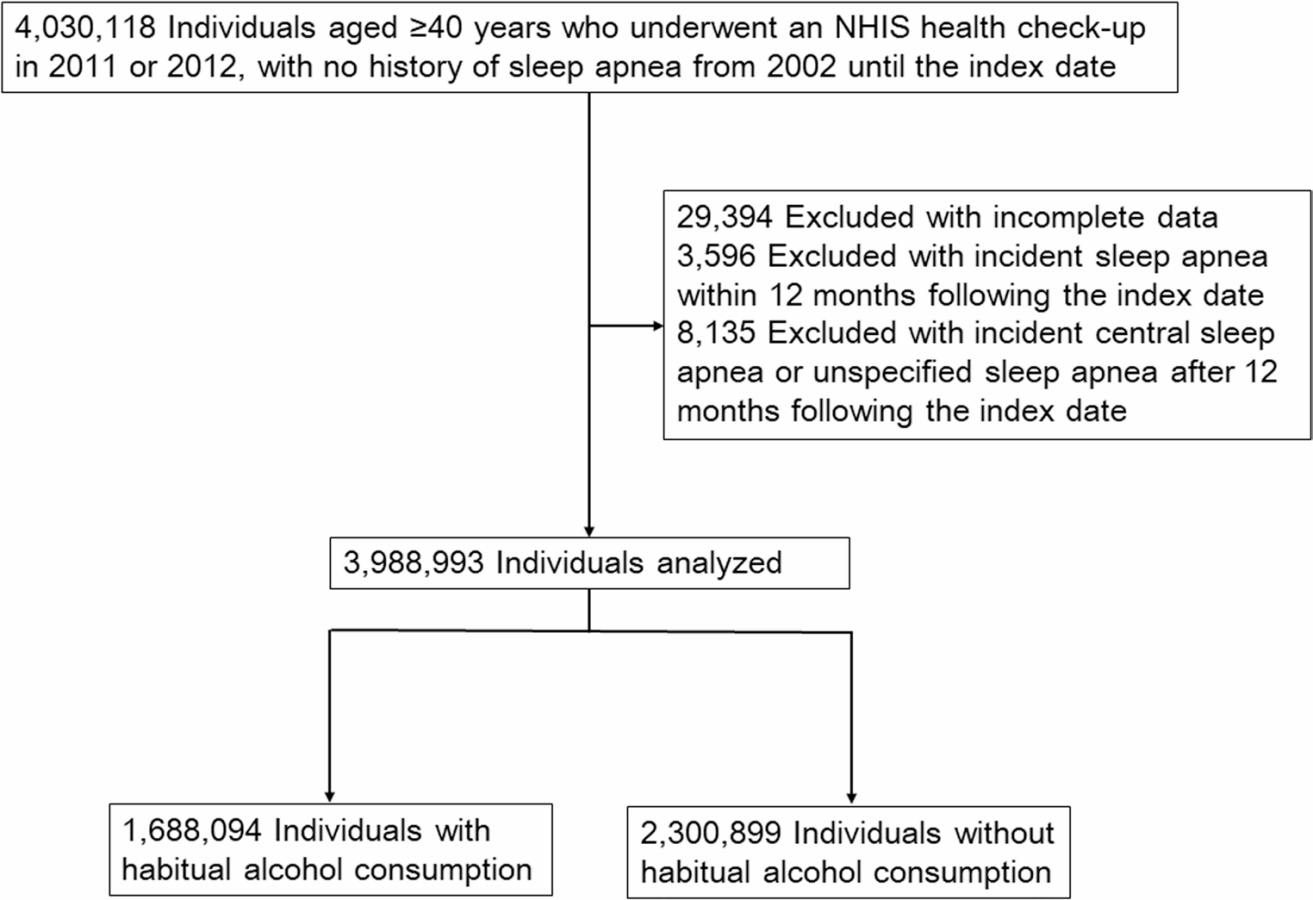
Flowchart of the study participants. The index date refers to the date on which participants underwent their health checkup. Definition of abbreviations: NHIS, National Health Insurance Services

A total of 4,030,118 individuals without a previous history of sleep apnea who participated in an NHIS health checkup in 2011 or 2012 were identified in the NHID. After excluding 29,394 individuals with incomplete data, 3,596 diagnosed with sleep apnea within 12 months of the index date, and 8,135 diagnosed with incident central sleep apnea or unspecified sleep apnea, the final analysis included 3,988,993 participants (Fig. 1). The participants had a median age of 53 years (IQR, 46–62) and a median BMI of 23.8 kg/m² (IQR, 21.8–25.8); 50.1% were male. Of these, 1,688,094 (42.3%) reported habitual alcohol consumption.

The baseline characteristics of the study population are presented in Table 1. The individuals with habitual alcohol consumption were significantly younger, with a median age of 50 years (IQR, 44–57) compared to 56 years (IQR, 48–64) in those without habitual alcohol consumption, and there was a higher proportion of males (76.2% vs. 31.0%) and a greater prevalence of obesity (36.9% vs. 33.4%). The proportion of smokers was significantly higher among individuals with habitual alcohol consumption (61.9% vs. 19.1%), and a history of smoking ≥ 30 pack-years was more common in this group (13.5% vs. 5.0%). Individuals with habitual alcohol consumption were more physically active, with a median MET of 510 min/wk (IQR, 180–890) compared to 370 min/wk (IQR, 0–740) in those without habitual alcohol consumption. The proportion of individuals with a CCI score of 2 or higher was lower in the habitual alcohol consumption group (25.4%) compared to the non-habitual alcohol consumption group (31.4%).

**Table 1:** Baseline characteristics of participants

Characteristic	Habitual alcohol consumption (*n* = 1,688,094)	No habitual alcohol consumption (*n* = 2,300,899)	*P* Value
Age, y	50 (44–57)	56 (48–64)	< 0.001
Age ≥ 55 y	538,007 (31.9)	1,190,233 (51.7)	< 0.001
Male sex	1,286,609 (76.2)	712,856 (31.0)	< 0.001
BMI, kg/m^2^	24.0 (22.1–26.0)	23.6 (21.6–25.7)	< 0.001
BMI ≥ 25 kg/m^2^	622,613 (36.9)	767,640 (33.4)	< 0.001
Height, cm	167 (161–172)	158 (153–165)	< 0.001
Weight, kg	66 (59–74)	59 (53–67)	< 0.001
Waist circumference, cm	83 (77–88)	80 (74–86)	< 0.001
Central obesity	376,814 (22.3)	511,482 (22.2)	< 0.001
Income			< 0.001
Q1–Q2	818,888 (48.5)	1,219,768 (53.0)	
Q3–Q4	816,971 (48.4)	1,007,079 (43.8)	
Unknown	52,235 (3.1)	74,052 (3.2)	
Smoking status			< 0.001
Never	642,672 (38.1)	1,861,253 (80.9)	
Former	444,825 (26.4)	221,139 (9.6)	
Current	600,597 (35.6)	218,507 (9.5)	
Smoking, pack-years			< 0.001
0	642,672 (38.1)	1,861,253 (80.9)	
> 0 to < 15	428,655 (25.4)	180,599 (7.8)	
≥ 15 to < 30	389,343 (23.1)	143,394 (6.2)	
≥ 30	227,424 (13.5)	115,653 (5.0)	
MET, min/wk	510 (180–890)	370 (0–740)	< 0.001
Physical activity level, MET-min/wk			< 0.001
inactive group (0)	667,554 (39.5)	1,272,601 (55.3)	
insufficiently active group (1–499)	365,471 (21.6)	335,964 (14.6)	
active group (500–999)	279,159 (16.5)	268,289 (11.7)	
highly active group (≥ 1,000)	375,910 (22.3)	424,045 (18.4)	
MVPA, times/wk	2.0 (0.0–4.0)	0.0 (0.0–3.0)	< 0.001
MVPA category, times/wk			< 0.001
Physically inactive (0)	667,554 (39.5)	1,272,601 (55.3)	
1–2	365,471 (21.6)	335,964 (14.6)	
3–4	279,159 (16.5)	268,289 (11.7)	
≥ 5	375,910 (22.3)	424,045 (18.4)	
Charlson comorbidity index	0.0 (0.0–2.0)	0.0 (0.0–2.0)	< 0.001
≥ 2	429,204 (25.4)	722,948 (31.4)	< 0.001
≥ 5	85,041 (5.0)	215,385 (9.4)	< 0.001
Comorbidities			
Hypertension	555,280 (32.9)	939,375 (40.8)	< 0.001
Type 2 diabetes	419,329 (24.8)	766,606 (33.3)	< 0.001
Ischemic heart disease	242,636 (14.4)	475,063 (20.6)	< 0.001
Heart failure	33,637 (2.0)	102,339 (4.4)	< 0.001
Transient ischemic attack or cerebral infarction	98,490 (5.8)	264,602 (11.5)	< 0.001
Atrial fibrillation or flutter	27,361 (1.6)	57,204 (2.5)	< 0.001
COPD	88,533 (5.2)	202,700 (8.8)	< 0.001
CKD	105,139 (6.2)	235,511 (10.2)	< 0.001
Any cancer	125,064 (7.4)	257,316 (11.2)	< 0.001
Fatty liver	283,337 (16.8)	371,170 (16.1)	< 0.001
Laboratory findings			
Systolic BP, mmHg	124 (115–133)	120 (110–131)	< 0.001
Diastolic BP, mmHg	80 (70–84)	76 (70–80)	< 0.001
Triglycerides, mg/dL	122 (83–183)	106 (75–153)	< 0.001
Cholesterol, mg/dL	196 (173–220)	196 (172–221)	0.805
HDL-cholesterol, mg/dL	53 (45–63)	53 (45–62)	< 0.001
LDL-cholesterol, mg/dL	112 (91–134)	117 (95–140)	< 0.001
Fasting blood glucose, mg/dL	96 (88–107)	94 (87–104)	< 0.001
Hemoglobin, g/dL	14.6 (13.5–15.5)	13.3 (12.5–14.3)	< 0.001
Creatinine, mg/dL	0.9 (0.8–1.1)	0.8 (0.7–1.0)	< 0.001
eGFR, mL/min/1.73m^2^	81.9 (72.0–93.5)	79.4 (69.0–92.7)	< 0.001

Data are presented as No. (%) or median (interquartile range), unless otherwise noted

Abbreviations: *BMI* body mass index, *BP* blood pressure, *CKD* chronic kidney disease, *COPD* chronic obstructive pulmonary disease, *eGFR* estimated glomerular filtration rate, *HDL* high-density lipoprotein, *LDL* low-density lipoprotein, *MET* metabolic equivalent of task, *MVPA* moderate to vigorous physical activity

Baseline characteristics stratified by sex are presented in Supplementary Table 1. Males had higher proportions of habitual alcohol consumption (64.3% vs. 20.2%) and smokers (69.9% vs. 4.4%). Physical activity levels were also higher among males, with a greater MET (490 min/wk vs. 370 min/wk) than among females. The proportion of individuals with a CCI score of 2 or higher was lower in males than in females (25.1% vs. 32.7%).

### Main outcome

During a mean follow-up period of 9.7 ± 1.4 years, 33,563 individuals were diagnosed with OSA. The mean interval from the index date to OSA diagnosis was 6.3 ± 2.6 years. The incidence rate was 108.9 per 100,000 person-years in the habitual alcohol consumption group and 69.6 per 100,000 person-years in the non-habitual alcohol consumption group.

**Fig. 2:**
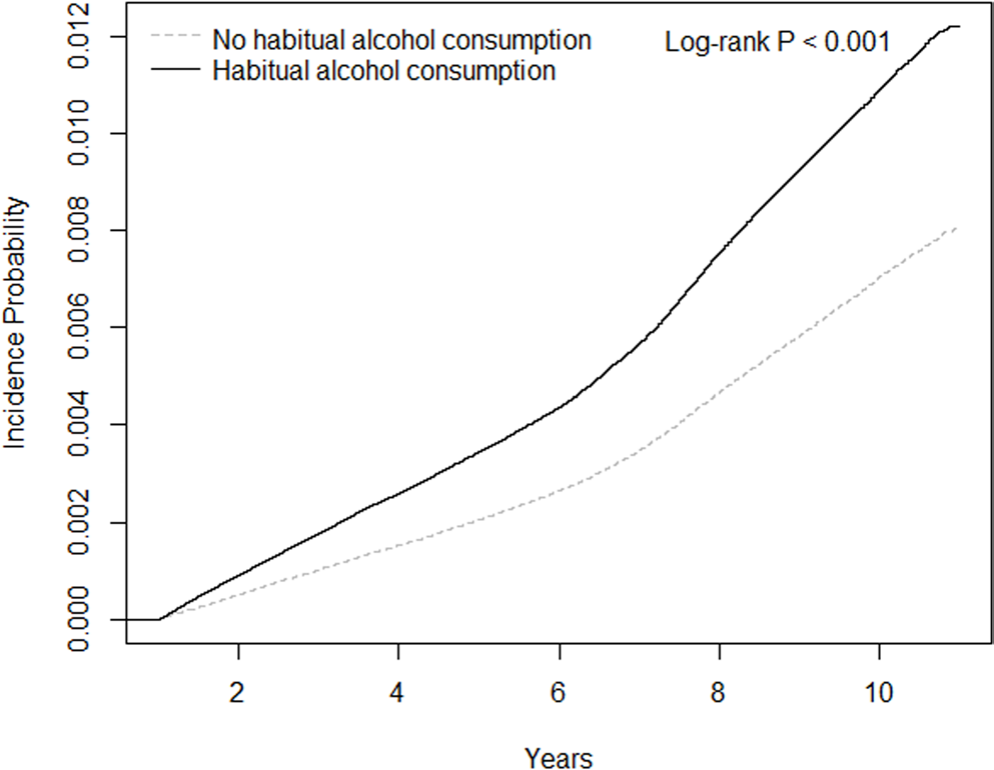
Kaplan–Meier curves of the incidence of obstructive sleep apnea according to habitual alcohol consumption

Kaplan–Meier curves of OSA incidence by alcohol consumption status at the index date are shown in Fig. 2. In an unadjusted Cox model, the habitual alcohol consumption group showed a higher risk of developing OSA (HR, 1.557; 95% CI, 1.524–1.590; Table 2). After controlling for potential confounders—including age, sex, BMI, income, smoking status, physical activity, and CCI—the association remained statistically significant, with an adjusted HR of 1.026 (95% CI, 1.001–1.051).

**Table 2:** Risk of incident obstructive sleep apnea by habitual alcohol consumption status

	*N*	Incident OSA	Follow-up duration, person-years	Incidence rate, per 100,000 person-years	Unadjusted HR (95% CI)	Adjusted HR (95% CI)*
Habitual alcohol consumption						
Yes	1,688,094	18,036	16,562,187	108.9	1.557 (1.524–1.590)	1.026 (1.001–1.051)
No	2,300,899	15,527	22,300,949	69.6	1	1

*Adjusted for age, sex, body mass index, income, smoking status, physical activity, and Charlson comorbidity index

Abbreviations: *CI* confidence interval, *HR* hazard ratio, *OSA* obstructive sleep apnea

## Subgroup analysis

The incidence rate of OSA was 125.5 per 100,000 person-years among males with habitual alcohol consumption, compared to 112.5 per 100,000 person-years among those without habitual alcohol consumption. The incidence rate of OSA was 56.2 per 100,000 person-years among females with habitual alcohol consumption and 51.5 per 100,000 person-years among those without habitual alcohol consumption (Supplementary Table 2). Kaplan–Meier curves showing OSA incidence based on habitual alcohol consumption status and sex are shown in Supplementary Fig. 1. After adjusting for age, BMI, income, smoking status, physical activity, and CCI, the HR for the incidence of OSA in males with habitual alcohol consumption was 1.039 (95% CI, 1.010–1.070). In contrast, the adjusted HR in females was 0.978 (95% CI, 0.931–1.027).

**Fig. 3:**
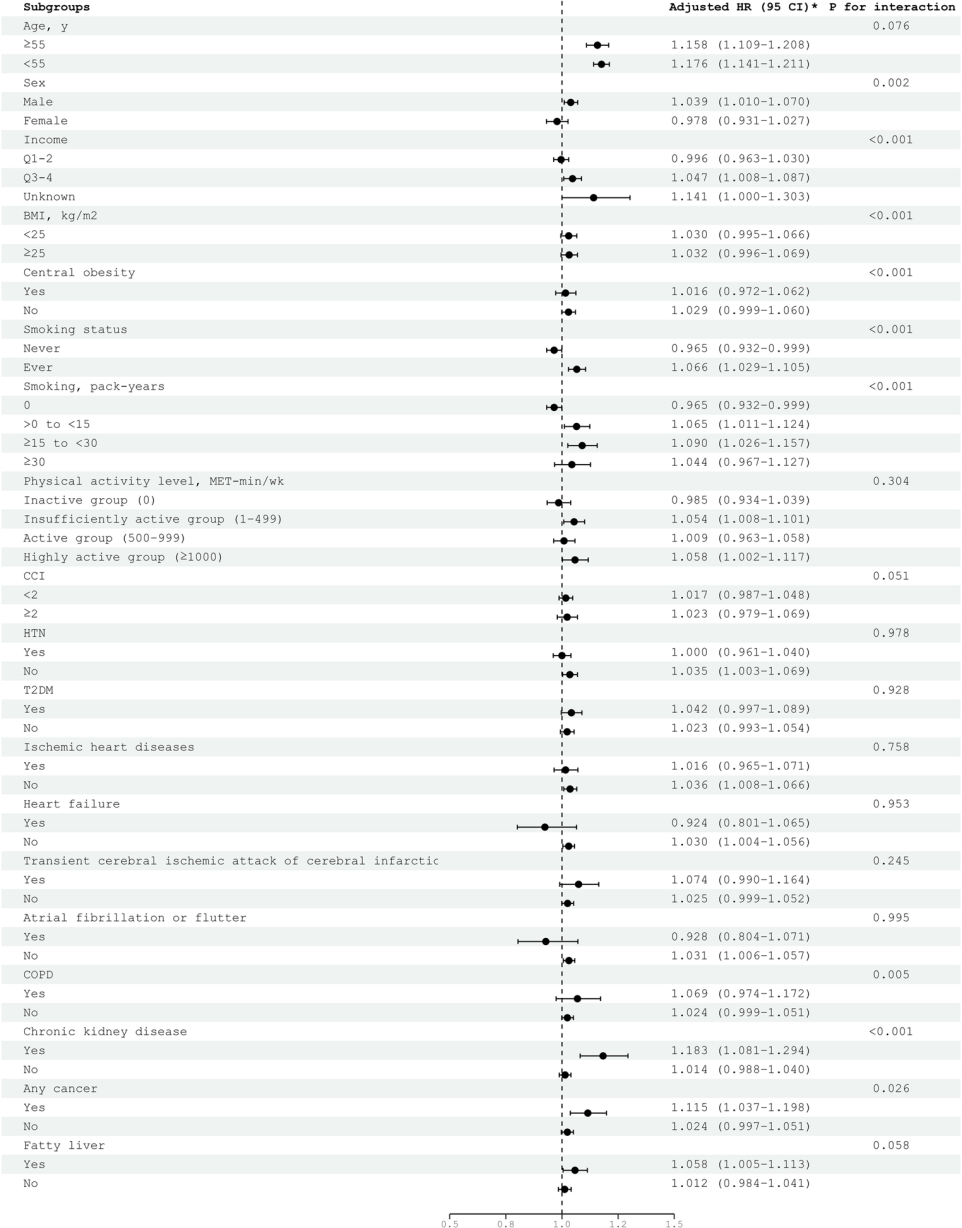
Subgroup analysis of the risk of incident obstructive sleep apnea by habitual alcohol consumption. *Adjusted for age, body mass index, income, smoking status, physical activity, and Charlson comorbidity index. Definition of abbreviations: BMI, body mass index; CCI, Charlson comorbidity index; CI, confidence interval; COPD, chronic obstructive pulmonary disease; HR, hazard ratio; HTN, hypertension; MET, metabolic equivalent of task; T2DM, type 2 diabetes

The results of the subgroup analyses based on the individual’s age, income, BMI, central obesity, smoking status, physical activity, CCI, and comorbidities are presented in Fig. 3 and Supplementary Table 3. The association between habitual alcohol consumption and incident OSA was more prominent in subgroups with CKD (adjusted HR, 1.183; 95% CI, 1.081–1.294) and a history of cancer (adjusted HR, 1.115; 95% CI, 1.037–1.198).

## Discussion

This large-scale nationwide population-based cohort study aimed to evaluate the association between habitual alcohol consumption and the incidence of OSA. Our key finding is that habitual alcohol consumption was associated with a mild but statistically significant increase in the risk of incident OSA across the general population. Importantly, we observed a heterogeneity in this association, which was particularly prominent among males and those with underlying CKD or a history of cancer. These findings suggest a temporal relationship between alcohol use and OSA development over a 10-year follow-up period, offering longitudinal evidence that complements prior cross-sectional research.

Previous studies have investigated the association between alcohol consumption and OSA. For example, according to the cross-sectional analysis from the Wisconsin Sleep Cohort Study, an increment of one drink per day in alcoholic beverage consumption was associated with a 25% increase in the odds of sleep disordered breathing in men [5]. In a case-control study involving 793 Chinese, alcohol consumption was an independent risk factor for polysomnography-confirmed OSA (adjusted odds ratio [aOR], 2.03; 95% CI, 1.30–3.17) compared with those who did not consume alcohol, with more pronounced associations among women [9]. Another cross-sectional study analyzed 11,859 participants from the Korean National Health and Nutrition Examination Surveys [8]. In this study, frequency and quantity of alcohol consumed was associated with high risk of OSA determined by the Snoring, Tiredness, Observed apnea, high blood Pressure-Body mass index, age, Neck circumference, and Gender (STOP-Bang) questionnaire [24]: individuals who drank 2–3 times per week had an aOR of 1.92 (95% CI, 1.67–2.21) compared to non-drinkers, and consuming 10 or more drinks at a time was associated with a more than threefold increase in OSA risk (aOR, 3.08; 95% CI, 2.57–3.70) compared to those drinking ≤ 2 drinks [8]. There was a randomized controlled study where 21 habitual snorers consumed moderate alcohol or a placebo in random order before sleep. Alcohol intake led to a statistically significant increase in the mean apnea-hypopnea index (from 7.1 to 9.7 events/hour, *P* = 0.017) [7]. However, the two recent Mendelian randomization studies found no significant causal association between genetically predicted alcohol consumption and OSA [11, 12].

The inconsistency in the literature is largely attributed to methodological limitations inherent in cross-sectional or case-control designs, which cannot establish temporality and are susceptible to various biases. These designs are susceptible to recall and selection biases and often lack comprehensive adjustment for confounding factors, such as physical activity or comorbidities. In contrast, the current study is a large nationwide longitudinal dataset with approximately 4 million individuals and a follow-up period of 10 years. By evaluating incident OSA rather than prevalent disease, we were able to clarify the temporal relationship between habitual alcohol consumption and OSA development. Furthermore, the inclusion of comprehensive confounders, such as age, BMI, smoking status, physical activity, and various comorbidities, strengthens the internal validity of our findings. In our study, the higher baseline prevalence of comorbidities observed in the non-habitual alcohol consumers compared with the habitual alcohol consumers may be attributed to the higher median age in the non-habitual alcohol consumption group and the ‘healthy drinker bias’; healthy individuals tend to drink more than individuals with chronic diseases [25, 26]. To address this, we adjusted for the overall disease burden using CCI in addition to age, and our findings remained robust. We found a mild but statistically significant association between habitual alcohol consumption, even at non-binge levels, and increased OSA incidence, particularly in males and individuals with CKD or cancer, the high-risk groups for OSA [27–30].

The plausible pathophysiologic mechanisms by which alcohol consumption may contribute to the development or exacerbation of OSA are multifactorial. Alcohol reduces the tone of the upper airway dilator muscles, such as the genioglossus [31], increasing upper airway collapsibility during sleep. Furthermore, alcohol suppresses the arousal response to apneic events and requires greater negative inspiratory pressures to trigger arousal, prolonging the duration of apneas and hypopneas and increasing the severity of oxygen desaturation [32, 33]. These pathophysiologic changes increase susceptibility to obstructive events during sleep, particularly in individuals with existing anatomical or functional predisposing conditions.

When stratified by sex, a significant association was observed only in males (adjusted HR, 1.039), whereas no significant association was found in females (adjusted HR, 0.978). This finding is consistent with the higher prevalence and severity of OSA in males, potentially attributable to anatomical and hormonal factors: males generally have longer upper airways and greater central adiposity, which predispose to pharyngeal fat deposition and airway collapsibility, whereas female sex hormones, particularly progesterone, enhance ventilatory drive and upper airway muscle tone.

A clinically relevant finding was the stronger association between alcohol consumption and OSA observed in specific high-risk subgroups. In our analysis, individuals with CKD and cancer demonstrated the highest risks (adjusted HR, 1.183 and 1.115, respectively). Overnight rostral fluid shifts promote pharyngeal soft tissue edema and increase upper airway collapsibility in patients with CKD [34]. Cancer is characterized by systemic inflammation and metabolic alterations, as well as by treatment-related effects, all of which may heighten vulnerability to sleep-disordered breathing [29, 35]. Such systemic alterations may increase vulnerability to the physiological effects of alcohol on sleep and respiration, thereby amplifying its influence on the manifestation of OSA.

Several limitations of this study should be acknowledged. First, the diagnosis of OSA was based on ICD-10 codes in administrative claims data rather than confirmed by polysomnography, which may lead to underdiagnosis or misclassification. Recent epidemiological data from the Finnish population show that the annual incidence of OSA has increased significantly—from 99 per 100,000 persons to 564 per 100,000 persons between 2005 and 2019—due to increased awareness and detection [36]. This suggests that the incidence rate of 69.6 to 108.9 per 100,000 person-years in our study may reflect underdiagnosis of this disease in the general population. However, the diagnostic validity of using the ICD-10 code for OSA is well supported. According to a recent validation study in the Danish population, the ICD-10 code for OSA demonstrates a high positive predictive value of 93.8% (95% CI: 85.0–97.5%) [37]. Second, our operational definition of habitual alcohol consumption as any weekly intake (> 0 g/week) may have classified light drinkers with low consumption volumes into the habitual alcohol consumption group, while occasional drinkers who consume alcohol less than once a week were categorized into the non-habitual consumption group. This misclassification bias could have attenuated the observed association between alcohol consumption and OSA. Moreover, further research is needed to assess dose-response effects. Third, information on alcohol consumption was collected based on the participants’ current status in the NIHD (2011–2012). Consequently, the period preceding the index date as well as longitudinal data were not available to assess changes in drinking behavior over time. This temporal limitation may have led to exposure misclassification during the follow-up period. Fourth, details on the timing of alcohol consumption—such as whether drinking occurred close to bedtime versus during the daytime or on weekends—were not available. Fifth, we could not assess the severity of incident OSA, as clinical metrics such as the apnea-hypopnea index are not available in the NHID.

## Conclusion

In this large, nationwide, and population-based cohort study, we found that habitual alcohol consumption was associated with a mild but statistically significant increase in the risk of developing OSA. This association was particularly evident among men and individuals with CKD or a history of cancer. Given the frequent alcohol consumption and the health burden of OSA in the general population, an understanding of the potential influence of alcohol consumption on OSA may support health promotion efforts and clinical counseling practices.

## Supplementary Information

Below is the link to the electronic supplementary material.

Supplementary Material 1

## Data Availability

The data that support the findings of this study are from the Korean National Health Insurance Service (NHIS). Restrictions apply to the availability of these data, which were used under license for the current study, and so are not publicly available. Data are available from the NHIS (https://nhiss.nhis.or.kr) upon reasonable request and with permission.
