# Supplementary material for: The influence of habitual consumption of alcohol on the incidence of obstructive sleep apnea: a national population-based cohort study
Source: Sleep Breath. 2026 Apr 18;30(2):127. doi: 10.1007/s11325-026-03684-1 (PMC13091808; doi:10.1007/s11325-026-03684-1)

**The influence of habitual consumption of alcohol on the incidence of obstructive sleep apnea: A national population-based cohort study**

Jina Park^1,*^; Eunhye Bae^2,*^; Yong Jin Sim^3^, Jaeyoung Cho, MD, PhD^1,4^

^1^Division of Pulmonary and Critical Care Medicine, Department of Internal Medicine, Seoul National University Hospital, Seoul, Republic of Korea

^2^Division of Pulmonary and Critical Care Medicine, Department of Internal Medicine, Chung-ang University Gwangmyeong Hospital, Gyeonggi-do, Republic of Korea

^3^Department of Biostatistics and Computing, Yonsei University, Seoul, Republic of Korea

^4^Department of Internal Medicine, Seoul National University College of Medicine, Seoul, Republic of Korea

**Supplementary Table 1. Baseline characteristics of participants according to sex.**

| **Characteristic** | **Male (n =** **1,999,465)** | **Female (n =** **1,989,528)** | ***P* Value** |
| --- | --- | --- | --- |
| Age, y | 52 (45–61) | 54 (46–62) | <0.001 |
| Age ≥55 y | 825,633 (41.3) | 902,607 (45.4) | <0.001 |
| BMI, kg/m^2^ | 24.2 (22.3–26.0) | 23.4 (21.5–25.6) | <0.001 |
| BMI ≥25 kg/m^2^ | 769,813 (38.5) | 620,440 (31.2) | <0.001 |
| Height, cm | 169 (165–173) | 156 (152–159) | <0.001 |
| Weight, kg | 68 (62–75) | 57 (52–62) | <0.001 |
| Waist circumference, cm | 84 (80–89) | 77 (72–83) | <0.001 |
| Central obesity | 475,850 (23.8) | 412,446 (20.7) | <0.001 |
| Income |  |  | <0.001 |
| Q1–Q2 | 916,194 (45.8) | 1,122,462 (56.4) |  |
| Q3–Q4 | 1,017,215 (50.9) | 806,835 (40.6) |  |
| Unknown | 66,056 (3.3) | 60,231 (3.0) |  |
| Smoking status |  |  | <0.001 |
| Never | 601,179 (30.1) | 1,902,746 (95.6) |  |
| Former | 638,417 (31.9) | 27,547 (1.4) |  |
| Current | 759,869 (38.0) | 59,235 (3.0) |  |
| Smoking, pack-years |  |  | <0.001 |
| 0 | 601,179 (30.1) | 1,902,746 (95.6) |  |
| >0 to <15 | 538,354 (26.9) | 70,900 (3.6) |  |
| ≥15 to <30 | 520,946 (26.1) | 11,791 (0.6) |  |
| ≥30 | 338,986 (17.0) | 4,091 (0.2) |  |
| Weekly alcohol consumption, g/wk | 37.5 (0.0–112.5) | 0.0 (0.0–0.0) | <0.001 |
| Habitual alcohol consumption | 1,286,609 (64.3) | 401,485 (20.2) | <0.001 |
| MET, min/wk | 490 (120–880) | 370 (0–740) | <0.001 |
| Physical activity level, MET-min/wk |  |  | <0.001 |
| Inactive group (0) | 438,250 (21.9) | 549,824 (27.6) |  |
| Insufficiently active group (1–499) | 563,786 (28.2) | 596,754 (30.0) |  |
| Active group (500–999) | 571,958 (28.6) | 515,348 (25.9) |  |
| Highly active group (≥1000) | 425,471 (21.3) | 327,602 (16.5) |  |
| MVPA, times/wk | 2.0 (0.0–4.0) | 0.0 (0.0–3.0) | <0.001 |
| MVPA category, times/wk |  |  | <0.001 |
| Physically inactive (0) | 840,086 (42.0) | 1,100,069 (55.3) |  |
| 1–2 | 399,505 (20.0) | 301,930 (15.2) |  |
| 3–4 | 309,808 (15.5) | 237,640 (11.9) |  |
| ≥5 | 450,066 (22.5) | 349,889 (17.6) |  |
| Charlson comorbidity index | 0.0 (0.0–2.0) | 0.0 (0.0–2.0) | <0.001 |
| ≥2 | 501,588 (25.1) | 650,564 (32.7) | <0.001 |
| ≥5 | 131,428 (6.6) | 168,998 (8.5) | <0.001 |
| Comorbidities |  |  |  |
| Hypertension | 750,302 (37.5) | 744,353 (37.4) | <0.001 |
| Type 2 diabetes | 586,040 (29.3) | 599,895 (30.2) | <0.001 |
| Ischemic heart diseases | 352,737 (17.6) | 364,962 (18.3) | <0.001 |
| Heart failure | 55,344 (2.8) | 80,632 (4.1) | <0.001 |
| Transient ischemic attack or cerebral infarction | 162,400 (8.1) | 200,692 (10.1) | <0.001 |
| Atrial fibrillation or flutter | 45,500 (2.3) | 39,065 (2.0) | <0.001 |
| COPD | 148,018 (7.4) | 143,215 (7.2) | <0.001 |
| CKD | 153,564 (7.7) | 187,086 (9.4) | <0.001 |
| Any cancer | 194,781 (9.7) | 187,599 (9.4) | <0.001 |
| Fatty liver | 359,822 (18.0) | 294,685 (14.8) | <0.001 |
| Laboratory findings |  |  |  |
| Systolic BP, mmHg | 125 (117–134) | 120 (110–130) | <0.001 |
| Diastolic BP, mmHg | 80 (70–85) | 75 (70–80) | <0.001 |
| Triglycerides, mg/dL | 127 (88–189) | 100 (71–143) | <0.001 |
| Cholesterol, mg/dL | 194 (171–218) | 197 (174–223) | <0.001 |
| HDL-cholesterol, mg/dL | 50 (43–59) | 56 (48–66) | <0.001 |
| LDL-cholesterol, mg/dL | 113 (91–135) | 117 (95–140) | <0.001 |
| Fasting blood glucose, mg/dL | 97 (89–108) | 93 (86–102) | <0.001 |
| Hemoglobin, g/dL | 14.9 (14.1–15.7) | 12.9 (12.2–13.6) | <0.001 |
| Creatinine, mg/dL | 1.0 (0.9–1.1) | 0.8 (0.7–0.9) | <0.001 |
| eGFR, mL/min/1.73m^2^ | 81.0 (71.0–92.5) | 80.0 (69.3–93.2) | <0.001 |

Data are presented as No. (%) or median (interquartile range), unless otherwise noted.

Abbreviations: BMI, body mass index; BP, blood pressure; CKD, chronic kidney disease; COPD, chronic obstructive pulmonary disease; eGFR, estimated glomerular filtration rate; HDL, high-density lipoprotein; LDL, low-density lipoprotein; MET, metabolic equivalent of task; MVPA, moderate to vigorous physical activity.

**Supplementary Table 2. Risk of incident obstructive sleep apnea by habitual alcohol consumption status, stratified by sex.**

| **Sex** | **Habitual alcohol consumption** | **N** | **Incident OSA** | **Follow-up duration, person-years** | **Incidence Rate, per 100,000 person-years** | **Unadjusted HR**  **(95% CI)** | **Adjusted HR**  **(95% CI)*** |
| --- | --- | --- | --- | --- | --- | --- | --- |
| **Male** |  |  |  |  |  |  |  |
|  | Yes | 1,286,609 | 15,804 | 12,590,860 | 125.5 | 1.119 (1.089–1.151) | 1.039 (1.010–1.070) |
|  | No | 712,856 | 7,537 | 6,778,907 | 111.2 | 1 | 1 |
| **Female** |  |  |  |  |  |  |  |
|  | Yes | 401,485 | 2,232 | 3,971,326 | 56.2 | 1.088 (1.038–1.140) | 0.978 (0.931–1.027) |
|  | No | 1,588,043 | 7,990 | 15,522,042 | 51.5 | 1 | 1 |

*Adjusted for age, body mass index, income, smoking status, physical activity, Charlson comorbidity index.

Abbreviations: CI, confidence interval; HR, hazard ratio; OSA, obstructive sleep apnea.

**Supplementary Table 3. Subgroup analysis of the risk of incident obstructive sleep apnea by habitual alcohol consumption.**

| **Subgroup** | **Adjusted HR (95% CI)*** | ***P* Value for interaction** |
| --- | --- | --- |
| Age, y |  | 0.076 |
| ≥55 | 1.158 (1.109–1.208) |  |
| <55 | 1.176 (1.141–1.211) |  |
| Sex |  | 0.002 |
| Male | 1.039 (1.010–1.070) |  |
| Female | 0.978 (0.931–1.027) |  |
| Income |  | <0.001 |
| Q1–2 | 0.996 (0.963–1.030) |  |
| Q3–4 | 1.047 (1.008–1.087) |  |
| Unknown | 1.141 (1.000–1.303) |  |
| BMI, kg/m^2^ |  | <0.001 |
| <25 | 1.030 (0.995–1.066) |  |
| ≥25 | 1.032 (0.996–1.069) |  |
| Central obesity |  | <0.001 |
| Yes | 1.016 (0.972–1.062) |  |
| No | 1.029 (0.999–1.060) |  |
| Smoking status |  | <0.001 |
| Never | 0.965 (0.932–0.999) |  |
| Ever | 1.066 (1.029–1.105) |  |
| Smoking, pack-years |  | <0.001 |
| 0 | 0.965 (0.932–0.999) |  |
| >0 to <15 | 1.065 (1.011–1.124) |  |
| ≥15 to <30 | 1.090 (1.026–1.157) |  |
| ≥30 | 1.044 (0.967–1.127) |  |
| Physical activity level, MET-min/wk |  | 0.304 |
| Inactive group (0) | 0.985 (0.934–1.039) |  |
| Insufficiently active group (1–499) | 1.054 (1.008–1.101) |  |
| Active group (500–999) | 1.009 (0.963–1.058) |  |
| Highly active group (≥1000) | 1.058 (1.002–1.117) |  |
| Charlson comorbidity index |  | 0.051 |
| <2 | 1.017 (0.987–1.048) |  |
| ≥2 | 1.023 (0.979–1.069) |  |
| Hypertension |  | 0.978 |
| Yes | 1.000 (0.961–1.040) |  |
| No | 1.035 (1.003–1.069) |  |
| Type 2 diabetes |  | 0.928 |
| Yes | 1.042 (0.997–1.089) |  |
| No | 1.023 (0.993–1.054) |  |
| Ischemic heart diseases |  | 0.758 |
| Yes | 1.016 (0.965–1.071) |  |
| No | 1.036 (1.008–1.066) |  |
| Heart failure |  | 0.953 |
| Yes | 0.924 (0.801–1.065) |  |
| No | 1.030 (1.004–1.056) |  |
| Transient ischemic attack or cerebral infarction |  | 0.245 |
| Yes | 1.074 (0.990–1.164) |  |
| No | 1.025 (0.999–1.052) |  |
| Atrial fibrillation or flutter |  | 0.995 |
| Yes | 0.928 (0.804–1.071) |  |
| No | 1.031 (1.006–1.057) |  |
| COPD |  | 0.005 |
| Yes | 1.069 (0.974–1.172) |  |
| No | 1.024 (0.999–1.051) |  |
| CKD |  | <0.001 |
| Yes | 1.183 (1.081–1.294) |  |
| No | 1.014 (0.988–1.040) |  |
| Any cancer |  | 0.026 |
| Yes | 1.115 (1.037–1.198) |  |
| No | 1.024 (0.997–1.051) |  |
| Fatty liver |  | 0.058 |
| Yes | 1.058 (1.005–1.113) |  |
| No | 1.012 (0.984–1.041) |  |

*Adjusted for age, sex, body mass index, income, smoking status, physical activity, Charlson comorbidity index.

Abbreviations: BMI, body mass index; CI, confidence interval; CKD, chronic kidney disease; COPD, chronic obstructive pulmonary disease; HR, hazard ratio; MET, metabolic equivalent of task.

**Supplementary Fig. 1.** Kaplan–Meier curves of the incidence of obstructive sleep apnea according to habitual alcohol consumption in males (A) and females (B).

A


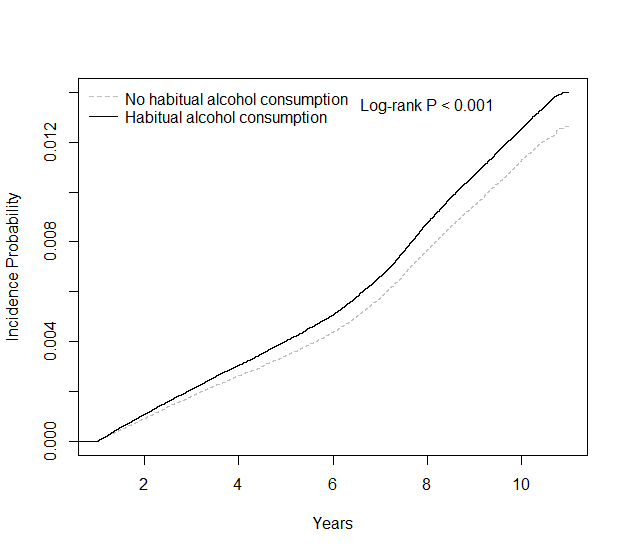


B


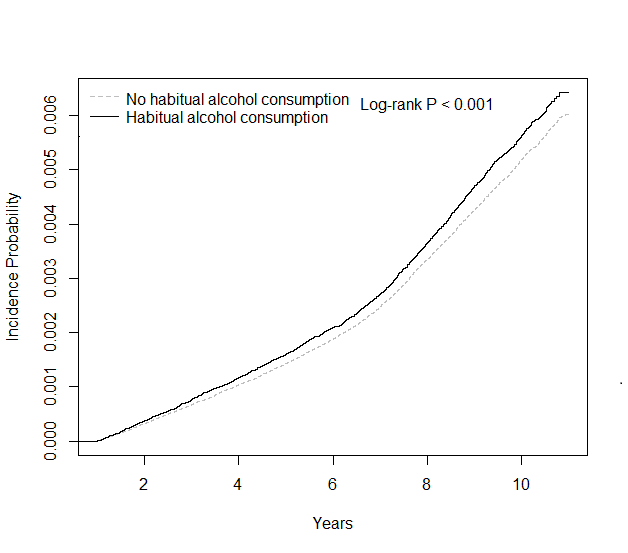

Supplement: Supplementary file 1 — Supplementary Material 1 [file 11325_2026_3684_MOESM1_ESM.docx]
